# NO, via its target Cx37, modulates calcium signal propagation selectively at myoendothelial gap junctions

**DOI:** 10.1186/1478-811X-12-33

**Published:** 2014-05-15

**Authors:** Kristin Pogoda, Monika Füller, Ulrich Pohl, Petra Kameritsch

**Affiliations:** 1 Walter Brendel Centre of Experimental Medicine, Munich Heart Alliance, Ludwig-Maximilians-Universität München, Munich, Germany; 2 DZHK (German Centre of Cardiovascular Research), partner site Munich Heart Alliance, Marchioninistr. 27, 81377 Munich, Germany; 3 Munich Cluster for Systems Neurology (SyNergY), Munich, Germany

**Keywords:** Nitric oxide, Connexin, Gap junction, Calcium, Myoendothelial

## Abstract

**Background:**

Gap junctional calcium signal propagation (transfer of calcium or a calcium releasing messenger via gap junctions) between vascular cells has been shown to be involved in the control of vascular tone. We have shown before that nitric oxide (NO) inhibits gap junctional communication in HeLa cells exclusively expressing connexin 37 (HeLa-Cx37) but not in HeLa-Cx40 or HeLa-Cx43. Here we studied the effect of NO on the gap junctional calcium signal propagation in endothelial cells which, in addition to Cx37, also express Cx40 and Cx43. Furthermore, we analyzed the impact of NO on intermuscle and on myoendothelial gap junction-dependent calcium signal propagation. Since specific effects of NO at one of these three junctional areas (interendothelial/ myoendothelial/ intermuscle) may depend on a differential membrane localization of the connexins, we also studied the distribution of the vascular connexins in small resistance arteries.

**Results:**

In endothelial (HUVEC) or smooth muscle cells (HUVSMC) alone, NO did not affect gap junctional Ca^2+^ signal propagation as assessed by analyzing the spread of Ca^2+^ signals after mechanical stimulation of a single cell. In contrast, at myoendothelial junctions, it decreased Ca^2+^ signal propagation in both directions by about 60% (co-cultures of HUVEC and HUVSMC). This resulted in a longer maintenance of calcium elevation at the endothelial side and a faster calcium signal propagation at the smooth muscle side, respectively. Immunohistochemical stainings (confocal and two-photon-microscopy) of cells in co-cultures or of small arteries revealed that Cx37 expression was relatively higher in endothelial cells adjoining smooth muscle (culture) or in potential areas of myoendothelial junctions (arteries). Accordingly, Cx37 - in contrast to Cx40 - was not only expressed on the endothelial surface of small arteries but also in deeper layers (corresponding to the internal elastic lamina IEL). Holes of the IEL where myoendothelial contacts can only occur, stained significantly more frequently for Cx37 and Cx43 than for Cx40 (endothelium) or Cx45 (smooth muscle).

**Conclusion:**

NO modulates the calcium signal propagation specifically between endothelial and smooth muscle cells. The effect is due to an augmented distribution of Cx37 towards myoendothelial contact areas and potentially counteracts endothelial Ca^2+^ signal loss from endothelial to smooth muscle cells. This targeted effect of NO may optimize calcium dependent endothelial vasomotor function.

## Background

More than 20 connexins have been described in mammalian cells so far [1], but only a few are expressed in blood vessels. In the endothelium Cx37, Cx40 and Cx43 are present whereas smooth muscle cells express Cx43 and Cx45 [2-5]. Although there is emerging evidence that connexins can exert channel-independent effects, e.g. in cell proliferation and migration [6,7], a main function of connexins consists of forming intercellular channels, the gap junctions [8]. Gap junctions (GJ) allow direct communication between cells by the exchange of ions and small signaling molecules like cyclic nucleotides [9]. Gap junctional communication (GJC) occurs between endothelial [10,11], as well as between smooth muscle cells [11-14]. In small vessels, gap junctions also connect endothelial and smooth muscle cells (myoendothelial gap junctional communication, MEGJC). Vascular GJC has an important impact on vascular function. Recently, we have shown that the endothelial Ca^2+^ responses to histamine and ATP are to a large extent dependent on an intact GJC. Blockade of GJC confines Ca^2+^ dependent NO production in endothelial cells in response to histamine stimulation to the relatively small population of EC that actually express histamine receptors [10]. Reduction of endothelial GJC in Cx40 knockout mice severely limited the spread of conducted dilation and induced atypic vasomotion with local vascular spasms [15]. Inhibition of GJC also decreased the vasodilation dependent on endothelial hyperpolarization (so-called EDHF-mediated dilation) [16,17]. Therefore, the regulation of GJ permeability and, hence, GJC can be considered an important feature in the control of vascular function and homeostasis. However, factors controlling vascular GJ permeability are not studied in great detail. Principally, it is known that pH [18], H_2_O_2_[19,20], hypoxia [21,22], and cannabinoids [23] reduce the intercellular communication. In previous experiments we characterized the role of NO on GJ permeability [24-26], demonstrating that NO can exert opposite effects, depending on the connexin that is expressed. In HeLa cells expressing only Cx40, NO increased, in a cAMP dependent manner the number of newly formed GJ containing Cx40 [24]. In contrast, in cells expressing only Cx37, NO acutely decreased the GJC by reducing the permeability of existing GJ [26] while no effect was observed in cells expressing only Cx43 [25]. Likewise, an inhibitory effect of NO on Cx37 containing GJ has also been shown in microvascular endothelial cells [27] and also in other cell types and species [28,29]. However, in these studies it remained unclear which connexins were affected [28,29].

In the present study we addressed the question whether the inhibitory effect of NO affects the overall GJ permeability when cells simultaneously express several connexins - in this case we focused on endothelial cells expressing the vascular connexins Cx37, Cx40 and Cx43. The GJ permeability was functionally assessed by the propagation of calcium signals (intercellular transfer of calcium or IP_3_) which is important in the endothelial control of vascular tone and cell adhesion. We also hypothesized that the inhibitory effect of NO on Cx37 containing GJ may be affected by a differential localization of Cx37 at membrane areas adjoining endothelial or smooth muscle cells. This was tested in co-cultures of endothelial and smooth muscle cells. Moreover, a distribution analysis of Cx was performed in intact small mesenteric arteries.

## Results

### Mechanically induced Ca^2+^_i_ increases propagated via gap junctions to neighbouring cells and were sensitive to NO in HeLa cells

**Figure 1:**
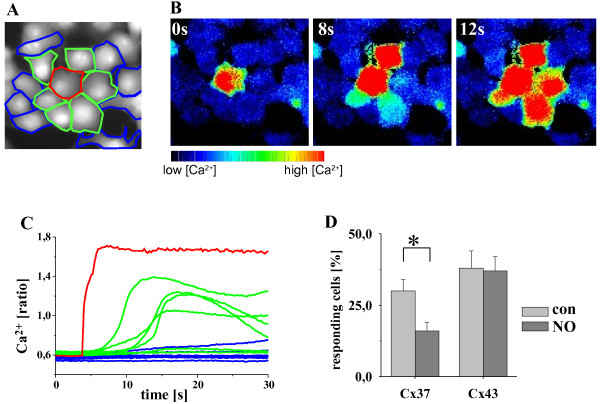
**NO reduced gap junctional Ca**^**2+ **^**signal propagation in HeLaCx37 cells. A** depicts the fluorescence image (Fura2, excitation 380 nm), the stimulated cell (red), adjacent cells (green) and secondary adjacent cells (blue). All adjacent cells together are counted as neighbouring cells. **B**. The Ca^2+^ concentration (ratio) increased after mechanical stimulation of the red marked HeLaCx43 cell (0 s) and the signal propagated to some neighbouring cells (8 s, 12 s). **C** depicts the time course of the Ca_i_^2+^ increase. The signal spread with a time delay of up to 7 s into adjacent (green, 1A green) but hardly to secondary adjacent (blue, 1A blue) cells. **D**. After stimulation, 30 ± 4% of the neighbouring HeLaCx37 cells showed a Ca^2+^_i_ increase (con). NO (15 min, 2 μM SNAP) reduced the number of cells responding with an elevated Ca^2+^_i_ signal to 16 ± 3% (p < 0.05, n = 15, w = C = 3) whereas it was virtually unchanged in HeLa-Cx43 cells (con: 38 ± 6%, NO: 37 ± 5%; n = 13, w = C = 3).

Touching a single HeLa cell transfected with either Cx37 or Cx43 (Figure 1A red cell) with a glass rod induced a rapid increase of Ca^2+^_i_ in the mechanically stimulated cell (Figure 1B, 0 s), which spread to most of the directly adjacent cells (AC, Figure 1A green; Figure 1B, 8 s) with a time delay of up to 10 s but virtually not to secondary adjacent cells (SAC, Figure 1A blue; Figure 1B 12 s). Calcium signal propagation to AC and SAC was defined as propagation to neighbouring cells. The time course of the Ca^2+^_i_ increase in the individual cells in a typical experiment is depicted in Figure 1C. Ca^2+^_i_ increased rapidly in the mechanically stimulated cell (red trace in Figure 1A corresponds to the calcium changes in the cell marked with a red line in the panel) followed by increases of the Ca^2+^ signal in most AC (green cells/traces) but virtually no SAC (blue cells/lines). Incubation of HeLaCx37 cells with a NO donor (SNAP, 2 μM, 20 min) significantly reduced the number of cells responding with an elevated Ca^2+^_i_ signal (con: 30 ± 4%, NO: 16 ± 3%; p < 0.05, n = 15, w = C = 3; Figure 1D). In the few cells that still showed a calcium signal propagation, there was no change in the time delay of the calcium signal propagation (con: 3.3 ± 0.6 s, NO: 3.1 ± 0.5 s). In HeLaCx43 cells, no effect of NO on the propagation of the Ca^2+^_i_ signal could be observed (con: 38 ± 6%, NO: 37 ± 5%; n = 13, w = C = 3, Figure 1D). To analyze the rectification of the Ca^2+^ signal propagation, additional experiments in HeLa co-cultures of HeLaCx37 and HeLaCx43 cells were performed. Stimulation of HeLaCx37 cells led to a signal propagation to 89 ± 5% (n = 12, w = 5, C = 3) of neighbouring HeLaCx43 cells. Similarly, stimulation of HeLaCx43 cells led to a signal propagation to 83 ± 5% (n = 12, w = 5, C = 3) of neighbouring HeLaCx37 cells.

### Mechanically induced Ca^2+^_i_ increases propagated NO-independently via gap junctions in HUVEC

**Figure 2:**
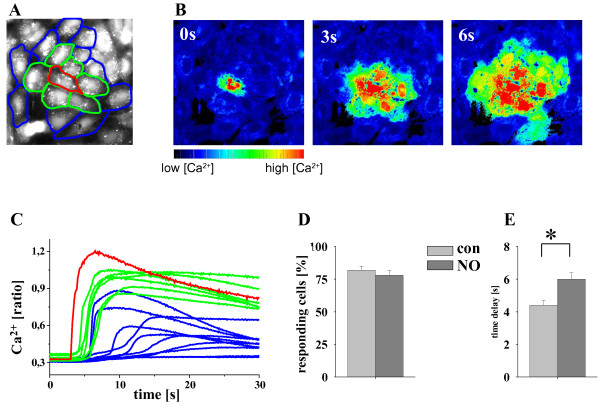
**NO did not reduce gap junctional Ca**^**2+ **^**signal propagation in HUVEC. A** depicts the fluorescence image (Fura2, excitation 380 nm), the stimulated cell (red), adjacent cells (green), and secondary adjacent cells (blue). All adjacent cells together are counted as neighbouring cells. **B**. The Ca^2+^ concentration (ratio) increased (0 s) after mechanical stimulation of the red marked HUVEC and the Ca^2+^_i_ signal propagated to most neighbouring cells within 6 s. The time course of the Ca^2+^_i_ increase in the marked cells **(A)** is shown in **C**. Ca^2+^_i_ increased in the stimulated cell (red) and the signal propagated with a time delay of up to 3 s to adjacent (green) and up to 10 s to secondary adjacent (blue) cells. In the HUVEC monolayer, incubation with NO (15 min, 2 μM SNAP) did not reduce the number of responding **(D)** but increased the time delay **(E)** of the Ca^2+^_i_ transfer to neighbouring endothelial cells. n = 39-66, w = 6, C = 4; *: p < 0.01, NG.

**Figure 3:**
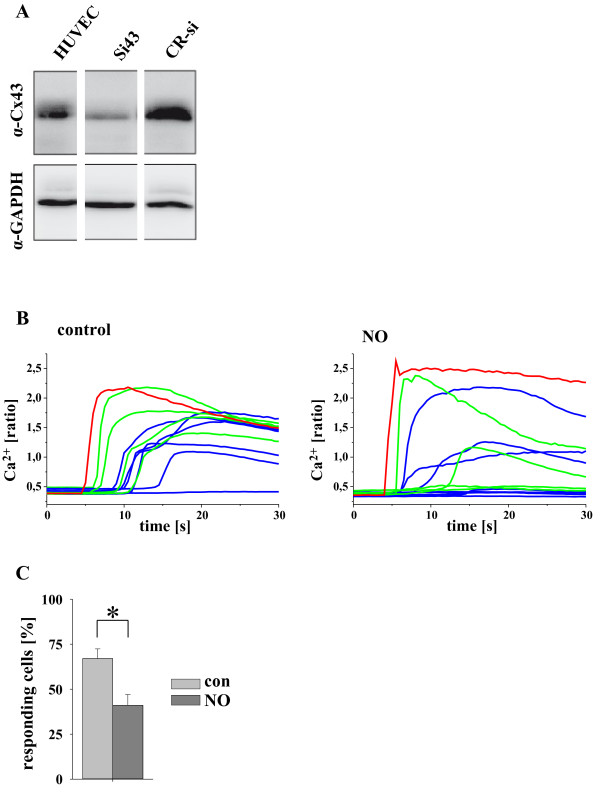
**NO reduced gap junctional Ca**^**2+ **^**signal propagation in siRNA (against Cx43) treated HUVEC. A**. Treatment with siRNA (24 h) against Cx43 decreased the amount of Cx43 protein in these cells. **B**. Depicts the time course of the Ca^2+^_i_ increase in the stimulated (red), adjacent (green) and secondary adjacent (blue) cells under control conditions (top) and after application of NO (bottom, 15 min 2 μM SNAP; representative experiment). The result of all experiments (n = 21, w = 6, C = 3; *: p < 0.05) is shown in **C**, demonstrating a decrease of responding cells after NO treatment.

In HUVECs, touching with a glass rod (Figure 2A, red cell) induced a rapid rise of Ca^2+^_i_ in the mechanically stimulated cell (Figure 2B, 0 s), which propagated to directly adjacent cells (AC, Figure 2B green; Figure 2B, 3 s) and from there to the secondary adjacent cells (SAC, Figure 2A blue; Figure 2B 6 s) with a time delay of 1–8 s. The time course of the Ca^2+^_i_ increase in the individual cells in a typical experiment is depicted in Figure 2C. Ca^2+^_i_ increased rapidly in the mechanically stimulated cell (red cell in Figure 2A corresponds with the red line in the panel) and the Ca^2+^ signal propagated to all AC (green cells/lines) and most of the SAC (blue cells/lines) with a time delay of 3 to 8 s, respectively. Calcium signal propagation to AC and SAC was defined as propagation to neighbouring cells. In contrast to HeLaCx37 cells, in HUVEC monolayers (expressing Cx37, Cx40 and Cx43), NO did not reduce the number of cells showing a Ca^2+^_i_ increase after mechanical stimulation of single cells (Figure 2D) but the time delay of the Ca^2+^_i_ onset in AC and SAC was significantly increased (con 4.4 ± 0.3 s, NO: 6.0 ± 0.4 s; n = 39-66, w = 7, C = 4; Figure 2E). Of note, NO did not affect the Ca^2+^_i_ increase in the initially stimulated cells (Δ ratio con: 0.89 ± 0.08, Δ ratio NO: 0.99 ± 0.07, n = 18, w = 7, C = 4).

### NO reduced the propagation of the Ca^2+^_i_ signals via GJ in HUVEC after downregulation of Cx43

Cx43 was downregulated in HUVECs by transfection with siRNA against Cx43. The amount of Cx43 protein in these cells was significantly reduced (to 40 ± 12%, n = 3) compared to non-silencing control siRNA as demonstrated by Western blot in parallel cultures to those used for the experiments (Figure 3A). Also Cx43 expression within the cell membrane was reduced after siRNA treatment whereas Cx37 expression and Cx40 expression was unchanged (Additional file 1: Figure S1). After knock down of Cx43, the number of neighbouring cells (AC and SAC) responding with an elevated Ca^2+^_i_ signal was significantly reduced by NO as depicted in the single traces of a representative experiment (Figure 3B con and NO). The mean results of 17 experiments are shown in Figure 3C revealing that the signal propagation from the stimulated cell to neighbouring cells was significantly reduced, although there was no difference in the maximal Ca^2+^_i_ increase of the stimulated cells (Δ ratio con: 1.25 ± 0.11, NO: 1.31 ± 0.09, n = 17, w = 6, C = 3, Figure 3B). Also, the maximal Ca^2+^_i_ increase and the time delay of the Ca^2+^ onset in neighbouring cells was unchanged in the remaining reacting cells (data not shown).

### In co-cultures of HUVEC and HUVSMC where Cx37 is highly expressed between both cell types, NO reduced myoendothelial coupling

**Figure 4:**
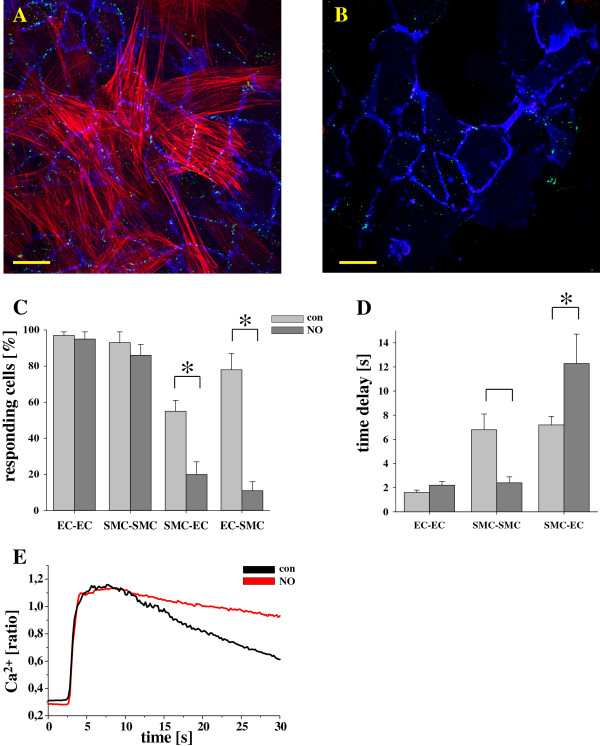
**NO inhibited myoendothelial signal propagation in co-cultured HUVEC and HUVSMC. A/B** depicts the immunohistochemical staining of Cx37 (EC, red), CD31 (EC, blue) and α-smooth-muscle actin (SMC, red) in a co-culture of endothelial and smooth muscle cells (**A**, scale bar 30 μm) and an area with endothelial cells only within the same co-culture (**B**, scale bar 30 μm). **C** depicts the number of responding cells in co-cultures of HUVEC and HUVSMC. Treatment with NO (15 min, 2 μM SNAP) significantly reduced the Ca^2+^_i_ signal transfer from SMC to EC and also from EC to SMC whereas it did not affect the signal transfer from EC to EC and from SMC to SMC (n = 13-21, w = C = 5; p < 0.05). In the remaining responding cells, the time delay **(D)** was significantly increased from SMC to EC whereas the signal spread faster from SMC to SMC after exposure to NO. The amplitude of the Ca^2+^_i_ increase (in the remaining responding cells) was unchanged in all cells (n = 17-106, w = 14-21, C = 6; *: p < 0.05, con vs. NO, NG). **E**. The decrease of the mechanically induced calcium rise in the initial stimulated cells was reduced by incubation with SNAP (15 min, 2 μM).

The location of Cx37 (green) in co-cultures of HUVEC (stained against CD31, blue) and HUVSMC (stained with the SMC marker α-smooth muscle cell actin, red) is depicted in Figure 4A/B. HUVEC showed a much higher Cx37 staining in areas that adjoined or overlapped with SMC (A) than HUVEC of the same culture which occasionally formed islands of “pure” EC (B). Quantitative evaluation of the specific Cx37 dependent fluorescence per cell (mean fluorescence intensity per EC in these islands in % of the respective mean fluorescence intensity per EC in areas where SMC and EC are overlapping) revealed that the mean Cx37 staining of EC was significantly reduced (58 ± 6%, n = 9 areas in 3 different co-cultures, p < 0.05) in EC remote from SMC compared to EC adjoining SMC (mean Cx37 staining per cell set to 100%). In these co-cultures in areas where EC and SMC were only partly overlapping (and red stained-EC and non-stained-SMC could be surely distinguished) the propagation of Ca^2+^ signals (Figure 4C) between endothelial cells only (EC-EC) or between smooth muscle cells only (SMC-SMC) was virtually unchanged after incubation with NO. In contrast, after stimulation of SMC, the propagation of the Ca^2+^ signal between SMC and EC was significantly reduced by NO. Similar data were found when HUVEC were stimulated and the signal propagation from HUVEC to SMC was analyzed. The time delay of the Ca^2+^_i_ onset (Figure 4D) after NO treatment was unchanged between EC whereas the signal propagation was significantly faster between SMC. In contrast, the Ca^2+^_i_ onset in remaining responding EC after stimulating a SMC was significantly delayed. The maximal Ca^2+^_i_ increase of the initially stimulated cells was unchanged by NO (Δ ratio EC: con: 1.00 ± 0.05, NO: 0.90 ± 0.15; Δ ratio SMC: con: 1.75 ± 0.15, NO: 2.05 ± 0.35; n = 18-22, w = 16-19, C = 6) however, the decrease of the calcium concentration in the stimulated cells (Figure 4E) was significantly slower (leading to a smaller descending slope) after NO treatment than under control conditions (con: -0.0233 ± 0,0051 , NO: -0.0034 ± 0.0008 [Δ ratio/s], n = 5-7, C = 3, p < 0.05). Additionally, after NO-treatment the calcium signal propagated to more adjacent EC from stimulated (SMC-adjoining) EC compared to control conditions (con: 44 ± 10%, NO: 76 ± 4%, n = 7-11).

### “Holes” of the internal elastic lamina in isolated small arteries stain significantly more frequently for Cx37 or Cx43 than for the other connexins

**Figure 5:**
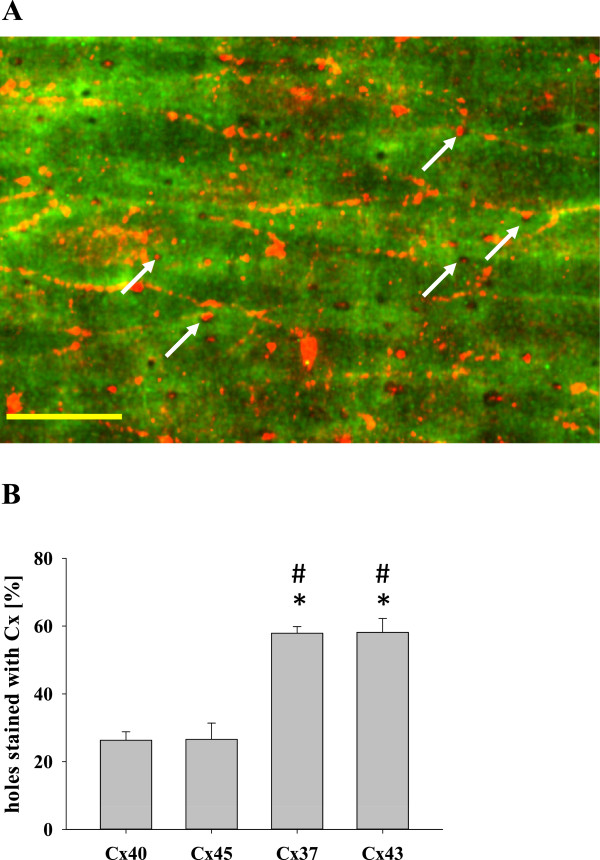
**Distribution of Cx37 within the internal elastic lamina. A**. Representative 2-photon-image of a small resistance artery for visualization of the internal elastic lamina and the connexins. Cx37 (red) located in the small holes (dark dots) within the internal elastic lamina (autofluorescence, green). Arrows indicate some of the holes in which Cx37 could be detected; scale bar: 25 μm. **B** depicts the summary of n = 7-10 experiments (3 vessels each) revealing the percentage of holes in the internal elastic lamina that contain the different vascular Cx (*: p < 0.05 vs. Cx40; #: p < 0.05 vs. Cx45, NG).

Figure 5A depicts the overlay of a z-stack in the xy-plane. Using a two-photon microscope, the auto-fluorescence signal of the internal elastic lamina (green) revealed small dark spots identified as “holes”, areas where the endothelial cells can make contact with smooth muscle cells. These holes were distributed homogeneously over the whole scanned area. Scanning for Cx37 (red) revealed that holes were frequently positive for Cx37 (Figure 5A). This was also the case for Cx43. In contrast, Additional file 2: Figure S2 depicts that Cx40 and Cx45 were rarely found in the holes. On the basis of the calculated percentage of holes within the internal elastic lamina exhibiting a staining for connexins (Figure 5B), we found that Cx40 as well as Cx45 was located in about 30% of the holes. In contrast, the percentage of holes showing positive staining for Cx37 as well as for Cx43 was significantly higher (about 60%), suggesting that predominantly endothelial Cx37 and smooth-muscle Cx43 are in contact in the area of the holes within the internal elastic lamina. To further analyze whether Cx37 or Cx43 were found more often in cell areas corresponding to holes, we used a second approach: Analyzing only holes that did not fall in line with the border of endothelial cells, we found in 33 ± 5% holes a positive staining for Cx37 but only in 4 ± 1% holes positive staining for Cx40. Shifting the region of interest (same size as holes) 6 μm apart from the holes (also not along the border of EC), only 7 ± 3% of these regions were positive for Cx37 and 2 ± 1% were positive for Cx40 (n = 6, 3 different vessels). In contrast, the overall staining of Cx37 and Cx40 at cell borders was comparable in all images (mean grey Cx37: 37 ± 4; Cx40: 46 ± 8).

### Location of vascular Cx across the wall of isolated small arteries

**Figure 6:**
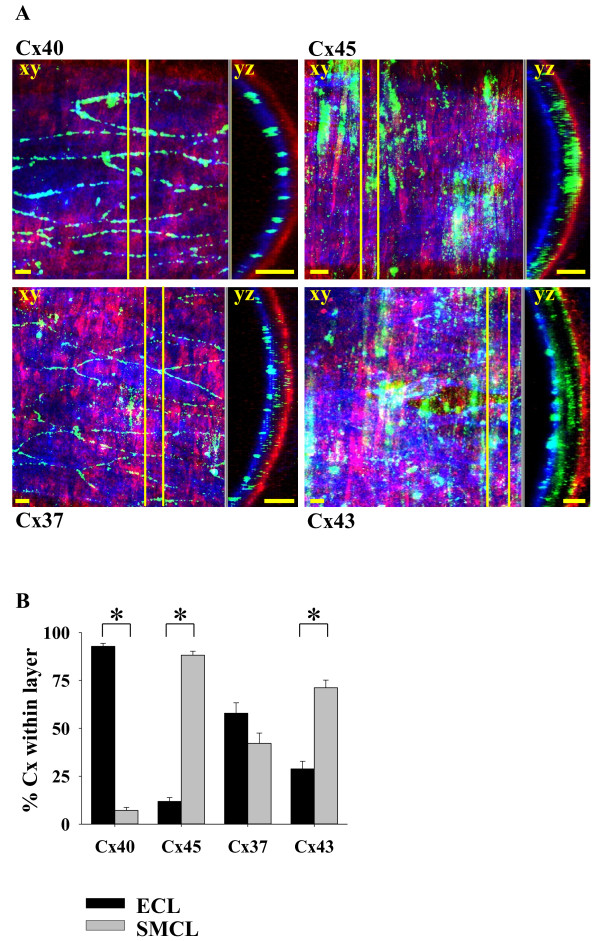
**Location of vascular Cx across the vessel wall. A**. Confocal images of triple (Cx, α-actin, CD31) immunohistochemical stainings of Cx40, Cx45, Cx37, and Cx43 in small resistance arteries. Left panel: Overlay of a z-series in xy-direction, right panel: Cross section (slice) of the z-stack in yz-direction along the yellow lines in the z-stack. The arrows depict the Cx expression in ECL (yellow) or beyond EC and within SMC (SMCL, white), scale bars: 10 μm. **B**. Summary of the Cx distribution within the ECL and SMCL for all Cx (n = 4-8, at least 3 vessels each; *: p < 0.001).

To reveal the distribution of connexins across the wall of intact small vessels, isolated arteries (with an outer diameter of about 200 μm) were stained with antibodies against the vascular connexins (Cx37, Cx40, Cx43, Cx45, green) and additionally with antibodies against CD31 (blue) and α-smooth muscle cell actin (red) to visualize the endothelial and smooth muscle cell layer, respectively. Figure 6A depicts the overlay of a z-stack in the xy-plane showing the distribution of Cx40 (green) within the vessel wall with an intact endothelial layer (CD31, blue) and smooth muscle cells (α-smooth muscle actin, red). A cross section in yz-direction (Figure 6A, Cx40, right panel) along the yellow lines shown in the z-stack reveals that Cx40 is indeed distributed only within the blue stained endothelial cell layer (yellow arrow). Additionally, vessels were stained against Cx45 (Figure 6A, Cx45) that is known to be expressed only in SMC. The staining of Cx45 (z-stack of xy plane) revealed – in contrast to Cx40 – a distribution exclusively along the SMC. The cross section in yz-direction (Figure 6A, Cx45 right panel) showed that Cx45 is indeed only present beyond the EC-layer and in the red stained SMC-layer (white arrow).

Similar to Cx40, Cx37 (Figure 6A, Cx37) was located along the borders of the endothelial cells (ECL). However, in contrast to Cx40, a spotted distribution of Cx37 was also found below the layer containing predominantly endothelial cells, i.e. closer to the layer containing predominantly smooth muscle cells (SMCL; Figure 6A, Cx37, right panel, white arrow) - a more precise separation being beyond the resolution limits of about 1–2 μm in z-direction. Likewise, Cx43 was not only located within the EC and SMC-layers only (Figure 6A, Cx43) but was also found between the respective outer borders of these cell layers (cross section in yz plane, Figure 6A, Cx43, right panel, yellow and white arrows). Calculating the percentage of Cx-staining within the ECL or SMCL (Figure 6B) revealed that Cx40 was mainly found within the endothelial layer whereas Cx45 was mainly distributed in SMCL. In contrast, about 60% of Cx37 staining was located in the layer of endothelial cells whereas about 40% of Cx37 staining was located below EC towards the SMCL. Similar to Cx37, Cx43 was found within and between both layers (ECL and SMCL): About 35% were located within the endothelial layer whereas 65% of the staining was located beyond the endothelial cells i.e. in SMCL and in between the layers.

## Discussion

Intercellular communication via gap junctions is considered pivotal for the functional coordination of cells within the vascular wall. It is based on the exchange of ions and other signaling molecules. The permeability and regulation of gap junction channels can vary considerably, depending on a) their differential composition by connexins and b) on their specific sensitivity to certain regulatory signals such as NO. Our findings are consistent with a significant role of NO and its target Cx37 in the control of calcium signal propagation between endothelial and smooth muscle cells (myoendothelial) but not between endothelial (inter-endothelial) or smooth muscle (inter-SM) cells, respectively. This implies a critical localization of the regulating mechanism or (and) of the target in the cellular regions where endothelial and smooth muscle cells adjoin. According to the data we obtained from cultured vascular cells and from freshly isolated small muscular arteries, a relative, local enrichment of Cx37 (as compared to other connexins) in the area of myoendothelial junctions plays a role for this NO effect on calcium signal propagation which occurs selectively in myoendothelial junctions. A selective inhibition of myoendothelial GJC (as opposed to inter-endothelial junctions) has already been described by Hu and Cotgreave [4]. In their experiments, LPS, TNF-α, and IL-1β caused a total inhibition of dye transfer between EC and SMC whereas it did not affect GJC between EC-EC and SMC-SMC. Although these authors did not investigate whether the inhibitory effect was due to NO, all compounds are known to increase endothelial NO production via induction of iNOS [4].

In a previous study in HeLa cells expressing only one type of connexins [26] we already showed that NO specifically reduces GJC when gap junctions contained Cx37. The GJC was analyzed by injecting a membrane-impermeable, but gap junction-permeable fluorescent dye (Alexa Fluor488) into a single cell and measuring its propagation to adjacent cells. Interestingly, the transfer of electric current was not inhibited by NO indicating that some ions could still pass the GJ though. In the present study we focused on the intercellular propagation of a calcium signal which may imply gap junctional passage of calcium itself or of a second messenger eliciting a calcium increase in the adjoining cells. We now provide evidence that the intercellular propagation of a calcium signal in vascular cells is also affected by NO however, surprisingly not in inter-endothelial gap junctions but in myoendothelial junctions only.

HUVEC, in contrast to the HeLa cells, contain not only one but usually three connexins (Cx37, Cx40 and Cx43, [2,5]). Their expression in the HUVEC used here has been confirmed by immunohistochemistry and Western blot previously [26]. It was similar to the expression we found in the isolated vessels. We have shown before in HeLa cells that NO has acute effects only on gap junctions containing Cx37 but not on gap junctions containing Cx43 or Cx40. The relative significance of Cx37 containing gap junctions for inter-endothelial calcium signal propagation was not very high since their blockade by NO only limited the speed of calcium signal propagation. This can be explained by the fact that all vascular connexins allow transfer of calcium signals via gap junctions [9,30] but are not all affected by NO. It is questionable whether the NO induced, slight delay of the calcium signal propagation in endothelial cells could have functional consequences with respect to calcium-dependent signaling processes. In general, however, an undisturbed inter-endothelial calcium signaling is necessary to enable a coordinated response of the endothelial layer to vasoactive agonists [10]. A clear reduction of calcium signal propagation in the endothelium could only be found when the Cx43 expression was substantially reduced. We suggest that this is due to an increased relative contribution of the other endothelial connexins Cx37 and Cx40 to gap junctional transfer whose expression was not affected by the knockout of Cx43. We did not knock down Cx40 in this context since we have shown before that NO’s augmenting effects on the permeability of gap junctions containing Cx40 [24] occur with a significant delay and play no role with regard to the acute effects of NO studied here.

In view of the compensating effect of the other connexins, at first glance, it appeared paradoxical that the NO effect was present in endothelial cells at all. A closer evaluation showed, however, that on a local level, the effect of NO was indeed important in endothelial cells. This effect was, however, restricted to areas, where endothelial cells adjoined smooth muscle cells. This can be considered as a form of functional compartmentalization of Cx37. Such a compartmentalization may be due to a differential distribution of the target mechanism or the target structure of NO, Cx37. In co-cultures, endothelial cells adjoining smooth muscle cells expressed more Cx37 than islands of “pure” endothelial cells in the same co-culture. Moreover, in the endothelium of intact vessels apart from endothelial cell-cell borders we found more holes of the IEL (representing potential areas of myoendothelial junctions outside endothelial cell borders) positively stained with Cx37 than in nearby control regions (apart from endothelial cell-cell borders and apart from holes) not directly adjoining smooth muscle. Interestingly, we found this enhanced location of Cx37 in only a fraction (about 30%) of the holes (apart from endothelial cell-cell borders) of the IEL. It is well known, however, that not every IEL-hole hosts myoendothelial junctions [31]. Indeed, the fraction observed in the arteries here is well in accordance with the fraction of IEL holes exhibiting morphologic evidence (by electron microscopy) for myoendothelial gap junctions in vessels of a similar type [31].

Sandow et al. [32] also found Cx37 within myoendothelial areas of rat mesenteric arteries. In addition, they identified Cx40 at the myoendothelial junctions. Using confocal microscopy we observed only a low expression of Cx40 in the area between endothelial and smooth muscle cells. Our results do not allow to decide whether the Cx distribution at the myoendothelial junctions we observed here reflects a typical static phenomenon for this type of vessel or whether it also reflects more a momentary state of dynamic redistribution of Cx. The latter cannot be excluded in view of the short half life time of connexins in the plasma membrane [33-35]. The observation reported in one study [31], that (probably in the same type of artery as studied here) there is morphological evidence for myoendothelial gap junctions in rats but not in mice, may be explained by such a dynamic behaviour.

Isakson et al. [36] analyzed the myoendothelial localization of Cx using actin-bridges as markers for the region of the MEJ in mouse cremasteric arteries. While Cx45 was not found in these actin-bridges Cx40, Cx37 and Cx43 were expressed at different amounts depending on the analyzed vascular bed. Indeed, several studies performed in other vessel types have shown with elegant techniques, that not only Cx37 but also Cx40 can be involved in the control of gap junction dependent signal exchange between endothelium and smooth muscle cells [16]. However, an acute, NO dependent component of Cx40 regulation has not been described. Cx45 was found mainly in smooth muscle cells by us and others [14,37,38] and it remained unclear whether it contributed to myoendothelial junctions. We found similar quantities of holes in the IEL staining for Cx37 and Cx43 suggesting that endothelial Cx37 could interact predominantly with smooth muscle Cx43 though other combinations (e.g. heteromeric [39,40] interaction of Cx37 and Cx43 in endothelial connexons or homotypic Cx43 GJ) probably also exist to some extent. Supporting this conclusion our additional analysis of the distribution of connexins across the vascular wall revealed, that mainly Cx37 and Cx43 were present in deeper layers corresponding to the region of the internal elastic lamina.

Our studies, focusing on the role of NO and on its target Cx37 were not designed to elaborate the role of different connexins in the control of myoendothelial gap junction communication. Of note, a relative predominance of Cx43 homotypic gap junctions at myoendothelial contacts as found for example in isolated thoracodorsal arteries [41] may even lead to NO induced improvement of GJC, since Straub et al. showed that NO can increase myoendothelial coupling via Cx43 by nitrosylation of Cx43 at C271. We did not find immunohistochemical or functional evidence for such a mechanism in our co-cultures and isolated vessels. Therefore, the potential formation of homotypic Cx43 gap junctions might not have been sufficiently high or, alternatively our cells might have had a high activity of *S*-nitrosoglutathione reductase, an enzyme that counteracts the augmenting effect of NO on Cx43 by de-nitrosylation [41]. Our results in pure endothelial cell cultures seem to support such a conclusion since NO had even a slightly delaying effect on intercellular calcium signal propagation in the presence of Cx43. This suggests that there was not sufficient Cx43 dependent augmentation of gap junctional calcium signal propagation to outweigh the Cx37 inhibition under the influence of NO.

In co-cultures of HeLaCx37 and HeLaCx43 cells, there was no rectification of the signal propagation and in co-cultures of EC and VSMC, we found that a gap junctional propagation of calcium signaling occurred in both directions. This was not readily expected since the smooth muscle cell volume is much bigger so that it might be supposed to act as a sink for endothelium derived calcium ions diffusing via gap junctions. However, the increase in smooth muscle calcium does not necessarily reflect diffusion of endothelial calcium only. At least part of the signal propagation might be due to the GJ passage of a messenger inducing a calcium release in the host smooth muscle cell. Previously, we have obtained indirect evidence that in endothelial cells part of the calcium signal propagation especially over longer distances seems to involve an IP_3_ dependent mechanism [10]. While it is known that IP_3_ (similar as other calcium release inducing compounds such a ADP Ribose [42], can pass gap junctions [43], experiments performed by Isakson [44] suggest that IP_3_ dependent calcium signal propagation may be unidirectional at least at some myoendothelial junctions due to an asymmetric life time of IP_3_ and of IP_3_ receptor expression respectively.

At present, we do not yet have functional data in isolated vessels which would allow an evaluation of the potential physiologic role of the selective effect of NO in myoendothelial junctions. However aside from myoendothelial feedback mechanisms already well shown and discussed by others [41,45-47] the inhibition of myoendothelial communication would be an effective mechanism to preserve or prolong the elevated Ca^2+^ signal within the endothelium since it inhibits potential calcium losses towards the smooth muscle cells. Indeed, we have found that in the presence of NO, endothelial cells adjoining smooth muscle cells exhibited a significantly reduced decay of the calcium elevation after mechanical stimulation when calcium signal propagation towards the smooth muscle cells was blocked. For the same reason, the signal induced in these SMC-adjoining endothelial cells spread to more EC in their vicinity after NO treatment. This goes along with our observation, that calcium signal propagation between SMC (when elicited by SMC stimulation) was increased under the same conditions. It is tempting to speculate that the observed prolongation of endothelial calcium elevation due to blockade of myoendothelial gap junctions may indeed improve gap junction dependent calcium signal propagation in the endothelium which we have shown to be pivotal for the functional response of the endothelium to vasoactive agonists [10].

## Conclusion

Our data indicate that NO controls bidirectional vascular calcium signal propagation specifically at myoendothelial gap junctions containing Cx37. The specific inhibitory effect of NO on myoendothelial gap junction communication is due to a relative increase of Cx37 in areas where endothelium and smooth muscle cells adjoin as assessed by analysis of Cx distribution in co-cultures and in isolated small arteries. The functional “compartmentalization” of the NO effect within the endothelial membrane allows an effective modulation of calcium signal propagation between these two types of vascular cells. The inhibitory action of NO preserves and prolongs the elevated calcium signal in stimulated endothelium and thereby promotes a more extended calcium signal propagation within the endothelium. It is tempting to speculate, that as a consequence, the response of the endothelium to calcium-increasing vasoactive agonists may be enhanced.

## Material and methods

### Cell culture

*Human umbilical vein endothelial cells* (HUVEC) were prepared as previously described by Jaffe and co-workers [48], with minor modifications. In brief, endothelial cells were harvested by incubation and subsequent perfusion of the veins of freshly obtained human umbilical cords with PBS^-^ (Mg^2+^ and Ca^2+^ free phosphate-buffered solution, in mmol/L: 136.89 NaCl, 5.37 KCl, 0.84 HNa_2_O_4_P*2 H_2_O, 0.44 KH_2_PO_4_, 5.55 Glucose Monohydrat, 3.57 NaHCO_3_, pH 7.4) containing 0.18 U/ml collagenase A (Roche). The harvested cells were seeded onto 10 cm culture dishes (Greiner) and were kept in Dulbecco´s modified Eagle medium (DMEM; life technologies), supplemented with 20% fetal calf serum (FCS; Biochrom), 10 U/ml penicillin (Sigma Aldrich), 10 mg/ml streptomycin (Sigma Aldrich) and substituted with endothelial cell growth medium (Promocell) at a 3:1 dilution. Cultured HUVEC were maintained at 37°C and 5% CO_2_, and passaged once or twice before culturing them on glass cover slips, pre-coated with collagen (10 μg/ml collagen G in Hepes, Biochrom). Cells were used for experiments as soon as they reached confluence. The umbilical cords were anonymous (donors non-identifiable) and informed consent was given for their use. For the use of HUVEC the approval was granted by the university ethics review board. All experiments were approved by the ethic committee of the Medical Faculty of the Ludwig-Maximilians-University Munich.

*Human umbilical vein smooth muscle cells (HUVSMC)* were isolated from freshly obtained umbilical cords by isolation of small vein-sections. After washing in PBS (PBS^-^ containing 0.28 mmol/L MgSO_4_*7 H_2_O, 0.35 mmol/L MgCl_2_*6 H_2_O and 0.88 mmol/L CaCl_2_*2 H_2_O) the sections were cut open longitudinally and the endothelium was scraped off with a scalpel. Afterwards the sections were cut into small pieces (500 μm × 500 μm), put with face down of the luminal side of the vein into 6 cm culture dishes and incubated with SMC-growth medium containing DMEM supplemented with 10 U/ml penicillin, 10 mg/ml streptomycin and 10% (FCS) and smooth muscle cell growth Medium 2, (Promocell) in a ratio 4:1 at 37°C and 5% CO_2_. After about 5 days the vessel pieces were removed, the remaining sprouted cells were washed with PBS and again incubated at 37°C and 5% CO_2_. After the cells reached confluence they were passaged once or twice before culturing them on glass cover-slips, pre-coated with collagen. Cultured HUVSMC were characterized regularly by immunohistochemical staining (followed by FACS analysis) against α-smooth-muscle-cell actin and CD31 revealing more than 95% α-smooth-muscle-cell actin positive and CD31 negative cells.

*HeLa cells*, stably-transfected with (mouse) Cx37 or (rat) Cx43 [49], were a kind gift from Dr. Klaus Willecke (University of Bonn, Germany). Cells were cultured at 37°C and 5% CO_2_ on glass cover slips in DMEM, supplemented with 10% new born calf serum (Biochrom), penicillin (10 U/ml), streptomycin (10 mg/ml). For the stably Cx37- and Cx43-transfected cells puromycin (1 μg/ml, Sigma Aldrich) was added to the cell growth medium. Wild-type HeLa cells do not express detectable amounts of connexin proteins [2].

*Co-cultures of HeLaCx37/HeLaCx43 cells or HUVEC/HUVSMC:* For experiments with co-cultured cells, the HeLaCx37 (or HUVEC) were labeled with the red fluorescent dye PKH26 (Sigma Aldrich) according to the manufacturer´s instructions at room temperature for 5 min and subsequently washed three times with PBS. Efficiency of the cell labeling was controlled in pilot experiments immediately after the staining procedure. In all cases, 100% of the cells were positive for PKH26 fluorescence (λ_ex_ = 543 nm, λ_em_ ≥ 570 nm). Freshly trypsinized PKH26-labeled HeLaCx37 (or HUVEC) and non-labeled HeLaCx43 (or HUVSMC) were mixed at a ratio of 1:1 (HeLaCx37/HeLaCx43) or 10:1 (HUVEC/HUVSMC). The mixed cell populations were plated on glass cover slips and used for coupling experiments within 15–30 hours.

*Knockdown of Cx43 mediated by siRNA transfection of HUVEC* was done according to the manufacturer´s transfection protocol (HiPerfect, Qiagen). In brief, the cells were transfected with 10 nmol/L of a synthetic siRNA against human Cx43 (si43, target sequence: ATGCTTAGAGTGGACTATTAA, Qiagen). As a control 10 nmol/L of a non-silencing siRNA (siCtr: AATTCTCCGAACGTGTCACGT, Qiagen) or DMEM alone (mock-control) was used. 24 hours after transfection the cells were washed twice with HUVEC medium and used for further experiments.

### Induction and Measurement of the intracellular calcium increase

A Ca^2+^_i_ increase was induced mechanically by touching a single cell with a glass rod (tip diameter about 1 μM) under optical control. The potential Ca^2+^ increasing effect of ATP that is released due to mechanical stimulation, was blocked by incubation with the ATP degrading enzyme apyrase (50 U/ml; Sigma) and the efficiency of the inhibition was tested regularly by addition of ATP (50 μmol/L; Sigma), not inducing a Ca^2+^ increase any more. Additionally, control experiments in apyrase-treated HeLa cells transfected with an empty vector did only lead to a calcium increase in the stimulated cell (see [30]). The Ca^2+^_i_ rise was detected using the Ca^2+^ sensitive dye Fura2 (Invitrogen). Cells were incubated in DMEM at 37°C and 5% CO_2_ including 4 μM Fura2. After 1 hour, the cover slips carrying cultured cell monolayers (HUVEC, HUVSMC, HUVEC/HUVSMC co-cultures or HeLa cells) were mounted in a custom made chamber on a heated microscope stage. The cells were pre-incubated for 15 min at 37°C in a HEPES buffered saline solution, HEPES+ (in mmol/L: 125 NaCl, 3 KCl, 1.25 NaH_2_PO_4_ 2.5 CaCl_2_, 1.5 MgCl, 10 Glucose, 10 HEPES, pH 7.4) containing N^ω^-nitro-L-arginine (LNA, 30 μM, Roche) and superoxide dismutase (SOD, 50 U/ml, Roche) in order to inhibit endogenous NO-formation and to scavenge superoxide anions.

The Ca^2+^ signal was detected by exciting the cells at 340/380 nm and measuring the fluorescence signal at 505 nm. Using a computerized system (Till Photonics) that controlled excitation wavelengths and camera settings, images of the analyzed areas (320 μm × 240 μm) were stored (frequency: 4 Hz, duration: 50 s) for each excitation wavelength. After each experiment the signal ratio (340 nm/380 nm corrected for the background was calculated for each pixel. The maximal Ca^2+^_i_ increase of single cells was determined by the maximal difference of the mean value of the ratio (calculated by the software for individual cells) before and after stimulation (Δ ratio). The change of the mean value of the ratio (x-axis) depicts the Ca^2+^ increase (y-axis) of these cells over time. The time delay between the Ca^2+^ onset of two cells was calculated by the difference of the time-points when the Ca^2+^ increase was at least 20% higher than the baseline in both cells. A cell was counted as a responding cell, when the ratio of the cell (reflecting the Ca^2+^_i_ concentration) increased more than 0.025. Beyond this value the signal to noise ratio was too low to distinguish between noise and signal. The exact amount of intracellular free calcium was not calculated.

*The effect of nitric oxide (NO)* was analyzed by incubation of the cells with HEPES+ buffer containing SNAP (S-nitroso-N-acetyl-D,L-penicillamine, 1 μmol/L, 15 min) before the experiments commenced. For control experiments the cells were incubated for 15 min with HEPES + buffer.

### Immunohistochemical analysis of cultured cells

Co-cultures of HUVEC and HUVSMC were stained as previously described by Donaldson [50]. Cells were fixed for 10 min in PBS-buffered paraformaldehyde (3%) and permeabilized in 0.2% Triton-X100 (AppliChem) in PBS for 2 min. Non-specific binding sites were blocked by incubation with 1% bovine serum albumin (BSA; AppliChem) in PBS. The cells were incubated with primary antibodies: rabbit polyclonal anti-Cx37 (1.25 μg/ml, Invitrogen, Cat. No. 42–4400, or 2.5 μg/ml Alpha Diagnostics, Cat. No. Cx37A11-A) and mouse monoclonal anti-CD31 (3 μg/ml, Abcam, Cat. No. ab9409), all diluted in PBS containing 1% BSA overnight. After washing with PBS the cell were incubated with secondary antibodies (20 μg/ml chicken anti-rabbit IgG labeled with AlexaFluor 488 and 10 μg/ml goat anti-mouse IgG, labeled with AlexaFluor 633; Invitrogen) diluted in PBS-1% BSA for 1 hour. After washout with PBS (+1% BSA) the cells were incubated for 1 hour with the direct-labeled (Cy3) anti-α-smooth-muscle-cell-actin, (mouse monoclonal, 28 μg/ml, Sigma Aldrich, Cat. No. C6198) and afterwards washed twice with PBS. The expression of membrane-localized Cx37 was analyzed at excitation wavelength of 488 nm, α-smooth-muscle-cell-actin at 546 nm and the endothelial cell marker CD31 at 633 nm using confocal microscopy (Leica TCS SP5). Background staining controls (not shown) were performed for each experiment and employed only the secondary antibody in permeabilized cells. SMC alone did not show any Cx37 staining.

### Immunohistochemical analysis of isolated vessels

All experimental procedures were approved by the ethics Committee of the University Munich in accordance with the German animal protection law. The investigation conforms to the European Commission Directive 2010/63/EU. Parts of the lower saphenous artery (outer diameter about 200 μm, C57BL/6, male, 26 ± 0.5 g, killed by cervical dislocation) were dissected and freed of surrounding tissue and incubated in PBS at room temperature. The vessels were cannulated and pressurized before fixation for 1 hour in 3.7% formalin (in PBS). After washing twice, and perfusing the for 5 min with PBS the vessels were incubated and perfused for 2 hours with PBS containing 1% BSA and 0.3% Triton-X100, washed and perfused again with PBS-1% BSA and incubated overnight with the antibodies against Cx37 (10 μg/ml Alpha Diagnostics, Cat. No. Cx37A11-A), Cx43 (see above), Cx40 (3.3 μg/ml polyclonal rabbit anti mouse-Cx40, Alpha-Diagnostics, Cat. No. Cx40-A) or Cx45 (2.5 μg/ml polyclonal rabbit anti Cx45 c-Term, Zymed, Cat. No. 40–7000) and against CD31 (2.5 μg/ml monoclonal rat anti mouse-CD31, Abcam, Cat. No 56299; antibodies were applied from the endothelial and smooth muscle side). After washing twice and perfusing the vessel with PBS-1% BSA, the appropriate secondary antibodies (Invitrogen, labelled with Alexa Fluor 488 or 633, diluted in PBS-1% BSA) were applied from the endothelial side for 2 hours. After washout with PBS-1% BSA the vessels were incubated (from the smooth muscle side) for 2 hours with a direct-labeled (Cy3) anti-α-smooth-muscle-cell-actin, (14 μg/ml, see above) followed by washing twice with PBS. The amount of membrane-localized Cx was analyzed at an excitation wavelength of 488 nm, α-smooth-muscle-cell-actin at 546 nm and the endothelial cell marker CD31 at 633 nm using confocal (Leica TCS SP5) and two-photon microscopy (TriMScope, LaVision BioTec) including an Olympus BX 51 microscope and a tunable Ultra II Titanium-Saphire Laser (Coherent). Background staining controls were performed for each experiment by incubation with the secondary antibodies only, in permeabilized vessels.

***Calculation of the connexin distribution*** within the smooth muscle cell layer, the endothelial cell layer and between both layers was done by determination of the volume containing the positive staining of the smooth muscle cells followed by calculation of the content of Cx staining in this volume (software: Imaris, Bitplane). The percentage of Cx localized within “holes” was determined using Till Vision (Till Photonics). Therefore, clearly identificable holes were counted and the Cx-fluorescence within these holes was determined. Fluorescence 25% higher than the background was counted as holes containing Cx. Finally, the percentage of holes with and without Cx was calculated.

### Data presentation and statistics

Gaussian distributed data were analyzed using a *t*-test. Non-Gaussian (NG, see figure legends) distributed data were statistically analyzed by the non-parametric Mann–Whitney rank sum test for non-paired data (for multiple comparisons, the Kruskal-Wallis One Way Analysis of Variance on Ranks followed by pairwise multiple comparisons with Dunn's Method were used). For all data obtained, “n” refers to the number of measurements, “w” to the number of different wells and “C” refers to the number of different cultures or vessels (i.e., separate experiments) employed. Differences were considered significant at an error probability of less than 0.05 (p < 0.05).

## Abbreviations

NO: Nitric oxide; GJ: Gap junctions; GJC: Gap junctional communication; EC: Endothelial cells; HUVEC: Human umbilical vein endothelial cells; SMC: Smooth muscle cells; HUVSMC: Human umbilical vein smooth muscle cells; Cx: Connexin; Ca^2+^: Calcium; EDHF: Endothelium derived hyperpolarizing factor; AC: Adjacent cells; SAC: Secondary adjacent cells; SNAP: S-nitroso-N-acetyl-D,L-penicillamine; siRNA: Small interfering RNA; CD31: PECAM-1, endothelial cell marker.

## Competing interests

The authors declare that they have no competing interests.

## Authors’ contributions

KP and PK developed the study, designed and performed the experiments, analyzed the data, created the figures and wrote the manuscript. MF performed part of the experiments, analyzed the data and generated the corresponding figures. UP was involved in planning and coordinating the study, drafted the manuscript and revised it critically for important intellectual content. All authors have read and approved the final version of the manuscript.

## Supplementary Material

Additional file 1: Figure S1 Immunohistochemical images of reduced Cx43 (red) and unchanged Cx37/Cx40 expression in siRNA treated HUVEC. Click here for file

Additional file 2: Figure S2 Distribution of Cx40, Cx43 and Cx45 within the internal elastic lamina. Click here for file
