# Supplementary material for: NO, via its target Cx37, modulates calcium signal propagation selectively at myoendothelial gap junctions
Source: Cell Commun Signal. 2014 May 15;12:33. doi: 10.1186/1478-811X-12-33 (PMC4036488; doi:10.1186/1478-811X-12-33)
Supplement: Additional file 1: Figure S1 — Immunohistochemical images of reduced Cx43 (red) and unchanged Cx37/Cx40 expression in siRNA treated HUVEC. [file 1478-811X-12-33-S1.pptx]

## Slide 1
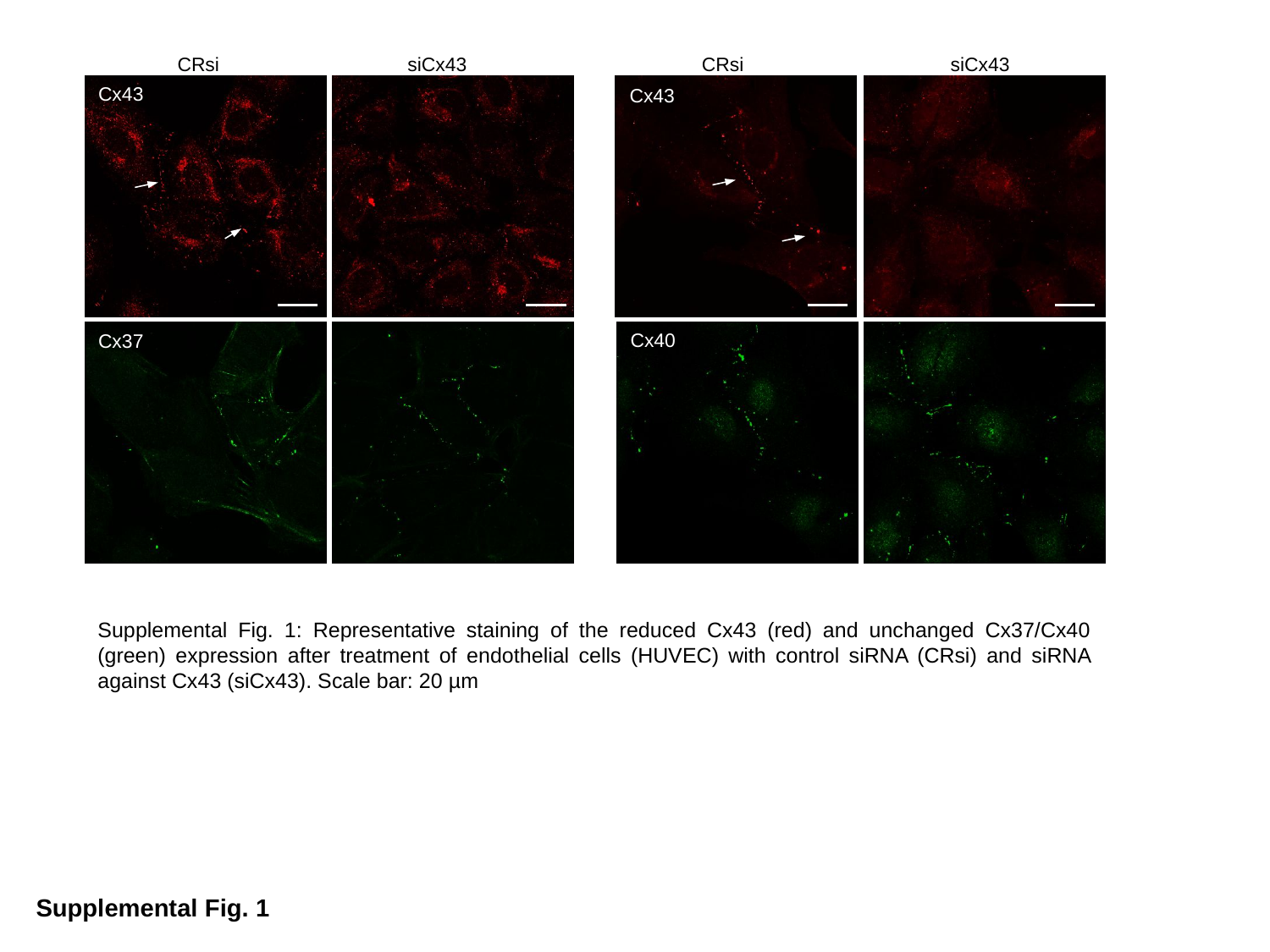

CRsi
siCx43
CRsi
siCx43
Cx43
Cx43
Cx40
Cx37
Supplemental Fig. 1: Representative staining of the reduced Cx43 (red) and unchanged Cx37/Cx40 (green) expression after treatment of endothelial cells (HUVEC) with control siRNA (CRsi) and siRNA against Cx43 (siCx43). Scale bar: 20 µm
Supplemental Fig. 1
