# Supplementary material for: NO, via its target Cx37, modulates calcium signal propagation selectively at myoendothelial gap junctions
Source: Cell Commun Signal. 2014 May 15;12:33. doi: 10.1186/1478-811X-12-33 (PMC4036488; doi:10.1186/1478-811X-12-33)
Supplement: Additional file 2: Figure S2 — Distribution of Cx40, Cx43 and Cx45 within the internal elastic lamina. [file 1478-811X-12-33-S2.pptx]

## Slide 1
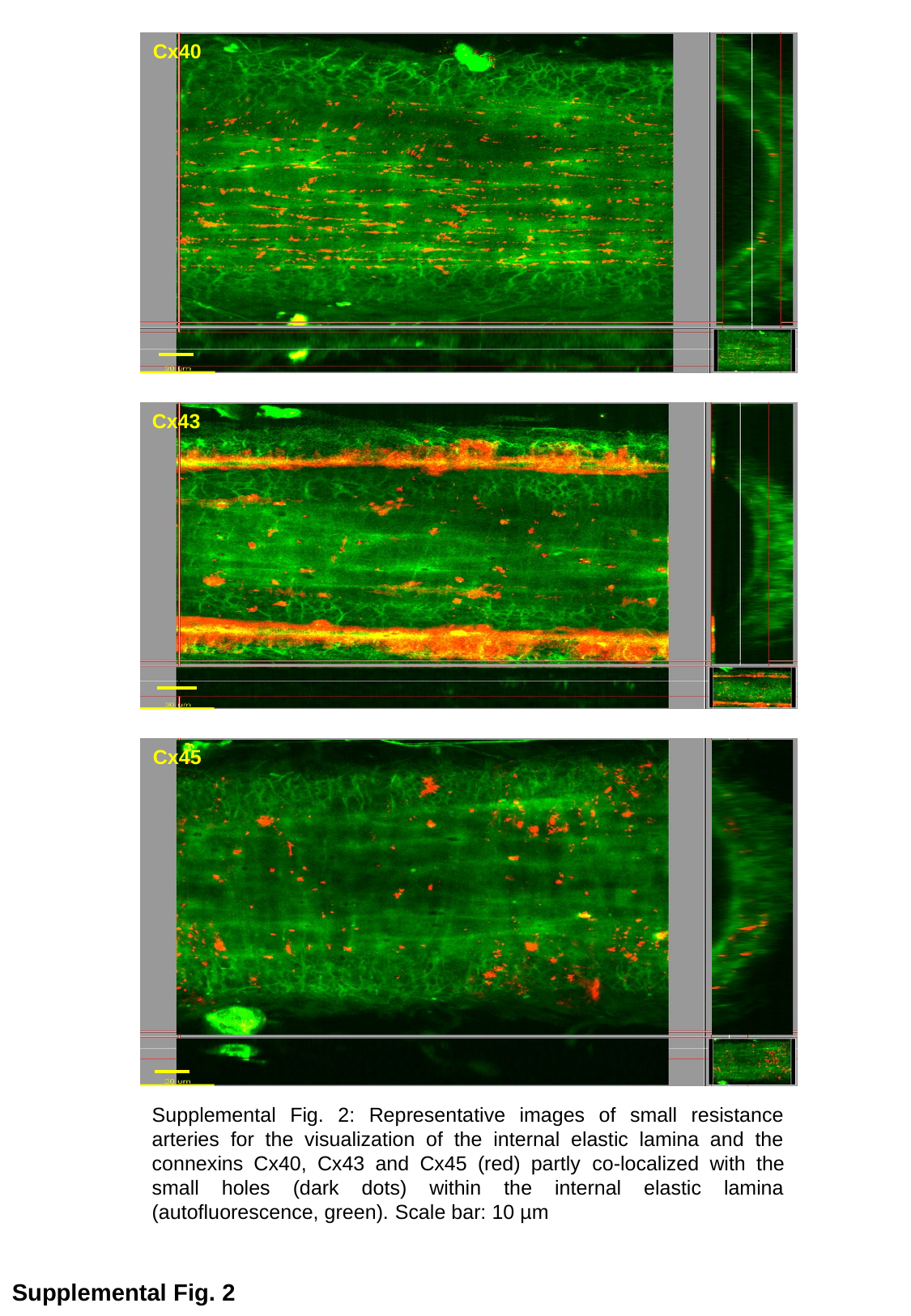

Cx40
Cx43
Cx45
Supplemental Fig. 2: Representative images of small resistance arteries for the visualization of the internal elastic lamina and the connexins Cx40, Cx43 and Cx45 (red) partly co-localized with the small holes (dark dots) within the internal elastic lamina (autofluorescence, green). Scale bar: 10 µm
Supplemental Fig. 2
